# Highly Pathogenic H5 Influenza Viruses Isolated between 2016 and 2017 in Vietnamese Live Bird Markets

**DOI:** 10.3390/v15051093

**Published:** 2023-04-29

**Authors:** Lizheng Guan, Gongxun Zhong, Shufang Fan, Erin M. Plisch, Robert Presler, Chunyang Gu, Lavanya Babujee, David Pattinson, Hang Le Khanh Nguyen, Vu Mai Phuong Hoang, Mai Quynh Le, Harm van Bakel, Gabriele Neumann, Yoshihiro Kawaoka

**Affiliations:** 1 Influenza Research Institute, Department of Pathobiological Sciences, School of Veterinary Medicine, University of Wisconsin, Madison, WI 53711, USA; 2 National Institute of Hygiene and Epidemiology, Hanoi 100000, Vietnam; 3 Department of Genetics and Genomic Sciences, Icahn School of Medicine at Mount Sinai, New York, NY 10029, USA; 4 Division of Virology, Department of Microbiology and Immunology, and International Research Center for Infectious Diseases, The Institute of Medical Science, University of Tokyo, Tokyo 108-8639, Japan; 5 Research Center for Global Viral Diseases, National Center for Global Health and Medicine, Tokyo 162-8655, Japan; 6 Infection and Advanced Research (UTOPIA) Center, The University of Tokyo, Pandemic Preparedness, Tokyo 108-8639, Japan

**Keywords:** influenza virus, H5, surveillance, Vietnam, deep sequencing, mice

## Abstract

Routine surveillance in live poultry markets in the northern regions of Vietnam from 2016 to 2017 resulted in the isolation of 27 highly pathogenic avian H5N1 and H5N6 viruses of 3 different clades (2.3.2.1c, 2.3.4.4f, and 2.3.4.4g). Sequence and phylogenetic analysis of these viruses revealed reassortment with various subtypes of low pathogenic avian influenza viruses. Deep-sequencing identified minor viral subpopulations encoding variants that may affect pathogenicity and sensitivity to antiviral drugs. Interestingly, mice infected with two different clade 2.3.2.1c viruses lost body weight rapidly and succumbed to virus infection, whereas mice infected with clade 2.3.4.4f or 2.3.4.4g viruses experienced non-lethal infections.

## 1. Introduction

Highly pathogenic H5N1 influenza viruses of the A/goose/Guangdong/1/96-lineage emerged in 1996 and have spread throughout Asia, Europe, the Middle East, and parts of Africa and North America, causing more than 890 laboratory-confirmed human infections with a case fatality rate of approximately 50% (https://www.cdc.gov/flu/avianflu/reported-human-infections.htm; accessed on 4 April 2023). These viruses have now evolved into 10 genetically distinct clades (clades 0–9) with multiple subclades.

In addition to their threat to public health, highly pathogenic H5 viruses have caused huge economic losses in the poultry industry. In Vietnam, highly pathogenic H5N1 influenza viruses were first reported in 2001 and were enzootic in poultry populations by the end of 2004. The early outbreaks in Vietnamese poultry populations were primarily caused by H5N1 viruses of clade 1. To control the spread of these viruses, mass poultry vaccination with a vaccine against a clade 1 H5N1 influenza virus was implemented in 2005 [1]. This vaccination campaign did not completely eradicate H5N1 viruses in Vietnam, and viruses of clade 1 continued to circulate in the southern part of the country where they evolved into subclades 1.1.1 and 1.1.2. In the central and northern regions of Vietnam, viruses of clade 2.3.2 were detected from about 2005 to 2008, and those of clade 2.3.4 were detected from about 2007 to 2010 (reviewed in [2]). In 2010, clade 2.3.2 viruses were re-introduced into Vietnam, and their descendants (that is, clade 2.3.2.1c viruses) spread rapidly throughout Vietnam in 2012 ([3,4,5]; reviewed in [2]).

Since their emergence, clade 2.3.4 viruses have undergone frequent reassortment with other avian influenza viruses, giving rise to viruses with NA genes of different subtypes, including H5N1, H5N2, H5N6, and H5N8 (reviewed in [6]). These viruses rapidly evolved into several genetically distinct clades. H5N6 viruses of clade 2.3.4.4 were first detected in Vietnam in 2014 [5] where they caused outbreaks in poultry and quail [7,8]. In recent years, outbreaks of highly pathogenic influenza in Vietnamese poultry have been caused predominantly by viruses of clades 1.1.2, 2.3.2.1c, and 2.3.4.4, with clade 1.1.2 viruses primarily detected in southern Vietnam, clade 2.3.2.1c viruses detected throughout the country, and clade 2.3.4.4 viruses mostly detected in the central and northern provinces of Vietnam. In addition to their impact on the poultry industry, clade 2.3.4.4 viruses of the H5N6 subtype have also caused more than 80 laboratory-confirmed cases of human infection (https://www.cdc.gov/flu/avianflu/reported-human-infections.htm; accessed on 4 April 2023).

Live bird markets play an important role in influenza virus reassortment and spillover into humans [9,10,11,12,13,14,15,16,17]. Here, we conducted routine surveillance in Vietnamese live bird markets from 2016 to 2017 and identified 27 highly pathogenic H5 influenza viruses from apparently healthy birds. Sequence and phylogenetic analysis revealed extensive reassortment with other avian influenza viruses. Moreover, we found that viruses from different clades differed in their pathogenicity in mice.

## 2. Materials and Methods

### 2.1. Virus Isolation and Identification

In 2016–2017, 1765 oropharyngeal and cloacal swab samples were collected from apparently healthy birds in live bird markets in Northern Vietnam by the National Institute of Health and Epidemiology of Vietnam as part of routine surveillance activities. The samples were placed in a transport medium (DMEM containing 0.15% BSA, 100 IU/mL penicillin-streptomycin, 0.5 μg/mL amphotericin B, 100 μg/mL gentamicin, 20 μg/mL ciprofloxacin, and 0.02 M HEPES) for further processing. 

First, we pooled the samples (up to 10 samples collected from the same species on the same day in the same location) into several batches and inoculated them into 9–11-day-old specific pathogen-free (SPF) eggs that were incubated at 35 °C for 24–48 h. The allantoic fluid was then tested for hemagglutination activity. For hemagglutination-positive batches, individual samples were inoculated into two SPF eggs each and tested for hemagglutination activity. Next, RNA was extracted from the hemagglutination-positive samples using the MagMAX^TM^-96 Viral RNA Isolation Kit (ThermoFisher, Waltham, MA, USA), and one-step reverse transcriptase polymerase chain reaction (RT-PCR) was performed using the Superscript III high-fidelity RT-PCR Kit (ThermoFisher, Waltham, MA, USA) and oligonucleotides to the conserved regions of the viral matrix (M) gene. The sequences of the primers are as follows: M-73F, 5′-GTCAGGCCCCCTCAAAGC; M-242R, 5′-CGTCTACGCTGCAGTCC. For influenza-positive samples, portions of the hemagglutinin (HA) and neuraminidase (NA) genes were Sanger-sequenced to determine the subtype. 

### 2.2. Viral Genomic Sequence Analysis

For all highly pathogenic H5 viruses, the complete genomic sequence was determined with RT-PCR-amplifying extracted RNA with a mixture of oligonucleotides F1 (5′-GTTACGCGCCAGCAAAAGCAGG), F2 (5′-GTTACGCGCCAGCGAAAGCAGG), and R1 (5′-GTTACGCGCCAGTAGAAACAAGG). The amplified double-stranded cDNAs were purified with 0.45× volume of AMPure XP beads (Beckman Coulter) and fragmented with acoustic shearing (0.5–1 μg) on a Bioruptor Pico sonicator (Diagenode) with an average fragment size of 150 bp. Library preparation for next-generation sequencing used end repair, phosphorylation, A-tailing, and adaptor ligation NEBNext DNA library prep modules for Illumina (New England Biolabs), which was followed by additional PCR amplification. 

The Illumina MiSeq demultiplexed reads were processed and assembled using the Iterative Refinement Meta-assembler (IRMA v. 1.0.2) [18]. The internal gene sequences were obtained from the primary assembly, whereas the HA and NA sequences were obtained from the secondary assembly. We used the default IRMA FLU parameters with the following exceptions: LABEL was used for read sorting, the residual assembly factor was set to 400 for the secondary assembly, and reference elongation was prevented. All sequence data were preprocessed, the IRMA was run, and downstream analysis was conducted using version v0.9.9 of a snakemake workflow available at https://github.com/IRI-UW-Bioinformatics/flu-ngs/releases/tag/v0.9.9 (accessed on 28 June 2022). Consensus sequences were generated with the IRMA.

### 2.3. Phylogenetic Analysis

Nucleotide sequences of the H5Nx (for HA), HxN1 (for N1-NA), HxN6 (for N6-NA), and HxNx (for PB2 and NS genes) viruses were downloaded from the NCBI Influenza Virus Database (www.ncbi.nlm.nih.gov) and the Global Initiative on Sharing Avian Influenza Data (GISAID; www.gisaid.org; Supplemental Table S1). The sequences were aligned using MAFFT (https://mafft.cbrc.jp/alignment/software/). Phylogenetic analysis was performed using the MEGA 7.0 software package, implementing the Tamura-Nei nucleotide substitution model and the maximum likelihood method. The tree topology was evaluated using 1000 bootstrap analyses. 

### 2.4. Virus Stock Generation and Titration

The isolated, highly pathogenic H5 viruses were inoculated into three SPF eggs each (100 μL of 10^4^–10^5^-fold dilutions of virus). Following incubation at 35 °C for 24–48 h, the allantoic fluids were collected and combined. The stock virus titers were determined by performing plaque assays in Madin–Darby canine kidney (MDCK) cells. 

### 2.5. Pathogenicity Studies in Mice

Six-week-old female BALB/c mice (Jackson Laboratory, Bar Harbor, ME, USA) were anesthetized with isoflurane and inoculated intranasally with 10^4^ plaque-forming units (PFU) of each virus in a volume of 50 μL. Control mice were inoculated with 50 μL of PBS. Body weight and mortality were monitored daily for 14 days. Infected mice were euthanized if they lost more than 25% of their initial body weight. 

### 2.6. Safety Statement

The isolation of the surveillance samples was conducted in an enhanced biosafety level 3 (BSL 3+) laboratory at the University of Wisconsin-Madison. The RNA inactivation protocol was approved by the University of Wisconsin-Madison’s Institutional Biosafety Committee (IBC) after conducting a risk assessment in the Office of Biosafety. All experiments were approved by the University of Wisconsin-Madison’s IBC. This manuscript was reviewed by the University of Wisconsin-Madison Dual Use Research of Concern (DURC) Subcommittee. This review was conducted in accordance with the United States Government September 2014 DURC Policy. The DURC Subcommittee concluded that the studies described herein do not meet the criteria for Dual Use Research of Concern (DURC).

## 3. Results

### 3.1. Isolation and Identification of Avian Influenza Viruses from Birds in Vietnam in 2016–2017

**Figure 1:**
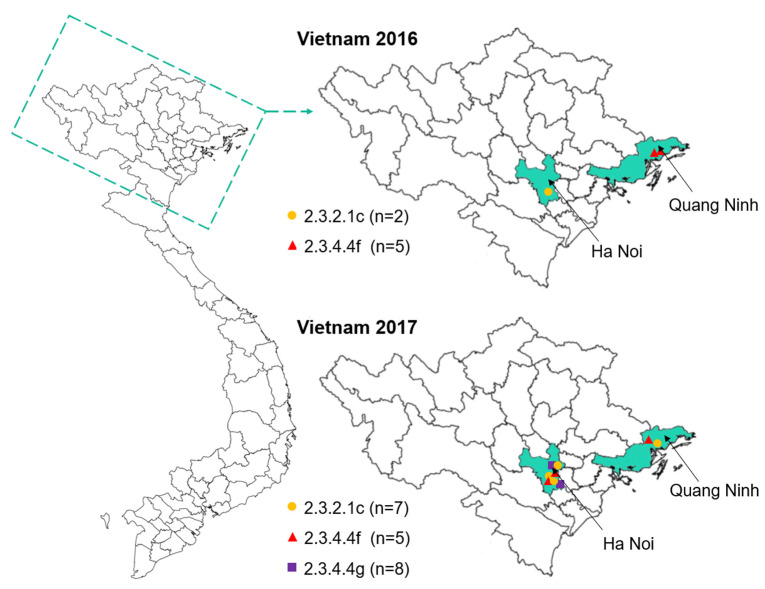
Geographic locations of surveillance activities in Vietnamese live bird markets from 2016 to 2017. Left, map of Vietnam with province borders. Right, magnification showing the northern part of Vietnam. The provinces where the samples were isolated are shown in aquamarine. The virus clades are indicated with different symbols; the number of isolates is listed in parentheses. The symbols in the map indicate the different locations at which viruses were collected.

We performed routine surveillance in live bird markets in several locations in northern Vietnam (Figure 1) by collecting oropharyngeal and cloacal swab samples from apparently healthy chickens, Muscovy ducks, and other ducks. In total, 1765 samples were pooled into batches of up to 10 samples (collected on the same day, in the same location, and from the same species), amplified in SPF-embryonated chicken eggs, and tested for hemagglutination activity. For hemagglutination-positive batches, samples were amplified individually and tested for their hemagglutination activity. Sanger sequencing of the viral HA and NA segments was carried out to determine the influenza virus subtype. We isolated 136 low pathogenic viruses of several subtypes including H1N1, H3N2, H3N6, H3N8, H4N6, H6N2, H6N6, H9N2, H9N6, and H11N2. Importantly, we also detected 27 highly pathogenic avian H5 viruses (Table 1), all of which were deep-sequenced. The deep-sequencing data revealed viruses of the H5N1 and H5N6 subtypes and several samples with ‘mixed’ sequences, indicating infection with more than one virus (Table 1).

### 3.2. Phylogenetic Analysis of Highly Pathogenic H5 Viruses

**Figure 2:**
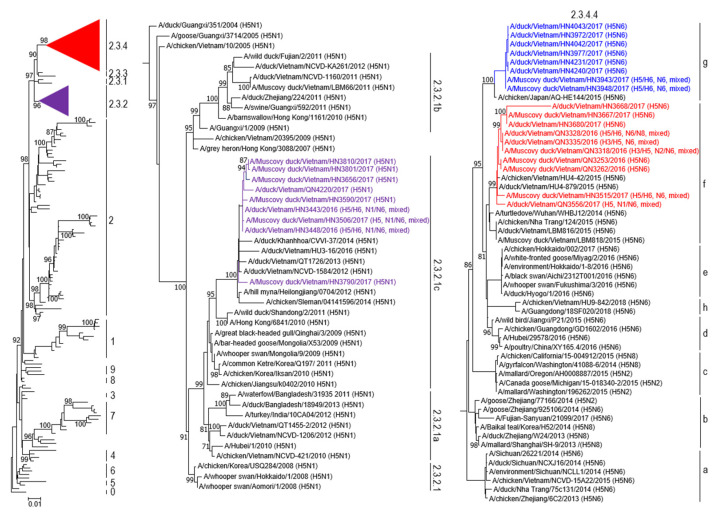
Phylogenetic analysis showing the HA genes of highly pathogenic avian H5N1 and H5N6 influenza viruses isolated in Vietnam from 2016 to 2017. Left panel: The phylogenetic tree of HA genes was rooted to A/goose/Guangdong/1/1996 (H5N1). Viruses of clades 2.3.2 and 2.3.4 were collapsed and are indicated by purple and red triangles, respectively. Details are shown in the center and right panels. The viruses sequenced in this study are marked in purple (clade 2.3.2.1c) or red and blue (clade 2.3.4.4). Center panel: Clade 2.3.2.1c viruses isolated in this study are shown in purple. Bootstrap values greater than 80% are indicated at the nodes. Right panel: Clade 2.3.4.4f and 2.3.4.4g viruses isolated in this study are shown in red or blue, respectively. Bootstrap values above 80% are indicated at the nodes.

To assess the genetic relationship of the isolated H5 viruses, we performed phylogenetic analysis for several viral genes (Figure 2 and Figures S1–S4). The HA genes belonged to clades 2.3.2.1c, 2.3.4.4f, and 2.3.4.4g of highly pathogenic H5 viruses of the A/Guangdong/1/1996-lineage (Figure 2). Most of the H5N1 viruses belonged to clade 2.3.2.1c and were closely related to H5N1 viruses previously detected in Vietnam. Viruses of the H5N6 subtype belonged to clades 2.3.4.4f and 2.3.4.4g. The clade 2.3.4.4f viruses were closely related to earlier Vietnamese H5N6 viruses, whereas the clade 2.3.4.4g viruses (all isolated in 2017) were more closely related to a Japanese H5N6 virus (Figure 2). 

The N1-NA genes of the H5N1 viruses shared 96.5–100% identity at the nucleotide level and were similar to H5N1 viruses previously isolated in Vietnam (Supplemental Figure S1). The N6-NA genes of the H5N6 viruses shared 94.8–100% identity at the nucleotide level and fell into two larger phylogenetic groups (Supplemental Figure S2). We also performed phylogenetic analyses for the PB2 and NS1 genes whose gene products contribute to pathogenicity. The PB2 genes of the H5 viruses showed distinct diversity and formed three groups in the phylogenetic tree (Supplemental Figure S3). The genetic diversity of the PB2 segments was consistent with earlier findings of extensive reassortment between highly pathogenic avian H5 viruses and low pathogenic avian influenza viruses of the H6N6, H9N2, and other subtypes [16]. The NS1 genes of the H5 viruses were found to belong to Allele A and shared 95.4–100% identity at the nucleotide level (Supplemental Figure S4); they were similar to other H5 viruses isolated in Vietnam between 2012 and 2019.

### 3.3. Genetic Analysis of Highly Pathogenic H5 Virus Amino Acid Sequences

First, we compared the consensus sequences of the isolated highly pathogenic H5 viruses (Supplemental Tables S2 and S3). The viral HA protein serves as the receptor-binding protein and is the major viral antigen. All H5 viruses analyzed here encoded HA-222Q/224G (numbering of mature HA protein used throughout), which is known to confer avian-type receptor-binding specificity [19]. The HA cleavage site motif (at which HA is post-translationally cleaved by host proteases into HA1 and HA2) of highly pathogenic avian influenza viruses contains multiple basic amino acids. In our study, the motif RERRRKR/G (the forward slash indicates the cleavage site) was detected in all H5 viruses except for A/Muscovy duck/Vietnam/HN3790/2017 (H5N1; clade 2.3.2.1c), which encoded RERRKR/G (Supplemental Tables S2 and S3). Previous studies have shown that loss of the 154–156 glycosylation motif may enhance binding to α2,6-linked sialic acids and virus transmissibility via respiratory droplets among mammals [20,21]. All H5 viruses characterized here lacked this glycosylation motif because they encoded alanine (A) at position 156; moreover, some of the viruses also lack an asparagine residue at position 154 (Supplemental Tables S2 and S3). The H5 subclades isolated here differed at several amino acid positions known to affect HA receptor-binding specificity, such as 189, 192, and 223 [22,23,24,25,26,27] (Supplemental Table S3); currently, the consequences of these differences for infection of mammals are not known. 

The viral NA protein encodes a sialidase that facilitates virus release from infected cells. Deletions in the NA stalk region are frequently observed in avian influenza viruses adapted to terrestrial birds and may enhance pathogenicity in mammals (reviewed in [28]). The N1-NA proteins analyzed here possessed a 20-amino acid deletion at positions 49–68 (Supplemental Table S2), which has been observed in the H5N1 influenza virus since 2000 and enhances its pathogenicity in mallard ducks and mice (reviewed in [28]). Some, but not all, of the N6-NA proteins analyzed here possessed a shorter deletion of 11 amino acids (positions 58–68) (Supplemental Tables S2 and S3), which increases virus replication in mammalian and avian cells [29]. 

NA is the target of several antiviral compounds that block its enzymatic activity. H5N1 viruses with H274Y or N294S (N1 NA numbering) substitutions in NA are resistant to oseltamivir [30,31]. Moreover, viruses encoding N6 NA-R292K (possessing a full-length NA stalk) show reduced susceptibility to zanamivir and laninamivir, whereas the N6 NA-E119V+I222L substitutions reduce the inhibitory effect of oseltamivir [32]. None of the H5N1 or H5N6 strains analyzed here encoded these substitutions, suggesting that they are sensitive to laninamivir and oseltamivir.

The PB2 protein is a component of the viral polymerase complex and an important determinant of the host range and pathogenicity of influenza viruses. Several amino acid changes can either promote virus replication and/or increase pathogenicity in mammals, including PB2-E158G, -T271A, -Q590S, -Q591K/R, -E627K, -D701N, and -S714R [33,34,35,36,37,38]. None of the viruses characterized here possessed amino acids associated with higher replicative ability in mammals or pathogenicity. Previously, we demonstrated that PB2-147T/339T/588T increased the pathogenicity of avian H5N1 viruses in mice [39]; these amino acids were detected in several of the clade 2.3.2.1c viruses (Supplemental Tables S2 and S3). In addition, most viruses analyzed here encoded PB2-588V, which increases viral polymerase activity and replication in cultured cells relative to viruses encoding PB2-588A [40]. Two viruses in clade 2.3.4.4g encoded PB2-199S (Supplemental Tables S2 and S3), which increases pathogenicity in mice compared with viruses encoding alanine at this position [41]. Moreover, one virus in clade 2.3.4.4f (A/Muscovy duck/Vietnam/HN3515/2017 (H5N6)) encoded PB2-526R, which was recently shown to enhance viral replication in cultured cells and pathogenicity in mice [42] (Supplemental Tables S2 and S3). 

Like PB2, substitutions with the viral PB1 polymerase protein can affect viral polymerase activity, including PB1-E180D and -M317V [43], PB1-T296R [44], PB1-V473L and -P598L [45], PB1-K480R [46], PB1-S524G [47], and PB1-K577E [48]. The viruses isolated in our study did not encode amino acids that would be expected to increase polymerase activity.

The PB1 viral RNA segment encodes a second protein, PB1-F2, which modulates the inflammasome, type I IFN responses, and the induction of apoptosis; its function also affects the severity of bacterial infection (reviewed in Refs. [49,50]). Most of the clade 2.3.2.1c and clade 2.3.4.4f viruses sequenced here encoded a premature stop codon, resulting in a truncated PB1-F2, as has been detected for other Asian H5 viruses isolated during this time period. In contrast, clade 2.3.4.4g viruses encoded full-length PB1-F2 proteins of 87 or 90 amino acids, respectively. A single amino acid substitution at position 66 (N66S) in PB1-F2 increases pathogenicity in mice [51]. One virus in clade 2.3.4.4f, A/Muscovy duck/Vietnam/QN3262/2016 (H5N6), encoded PB1-F2-66S, whereas all other clade 2.3.4.4 viruses encoded PB1-F2-66N (Supplemental Tables S2 and S3). 

The PA gene encodes another subunit of the viral polymerase complex. Several amino acid changes in PA have been reported to affect polymerase activity and/or pathogenicity in mice [52,53,54,55,56,57,58], but none of these amino acids was present in the viruses isolated here. However, all viruses analyzed possessed PA-383D, which confers increased polymerase activity to the H5N1 polymerase complex compared with a polymerase complex encoding PA-383N [59]. Most of the clade 2.3.4.4 viruses encoded PA-237E, which contributes to H5N1 pathogenicity in ducks [55] (Supplemental Tables S2 and S3), and all clade 2.3.2.1c viruses encoded PA-343S, which increases the pathogenicity of low pathogenic H5N1 virus in mice relative to a virus possessing PA-343A [57] (Supplemental Tables S2 and S3). 

The nucleoprotein (NP) is the main structural component of the viral ribonucleoprotein complex, and its interaction with host factors also contributes to the host range of influenza viruses [60]. A previous study found that the NP-M105V substitution increased the pathogenicity of an H5N1 virus in chickens [61]. In our study, all viruses in clade 2.3.2.1c and one virus in clade 2.3.4.4f possessed NP-105V; the remaining viruses encoded isoleucine or methionine at this position (Supplemental Tables S2 and S3). 

The matrix (M1) protein is the main structural component of influenza virions. H9N2 viruses of the G57 genotype are more pathogenic in chickens than H9N2 viruses of other genotypes, a phenotype that is conferred by the M1-37A, -95K, -224N, and -242N residues [62]. Several of the viruses characterized here encoded some of these amino acids, which may increase their pathogenicity (Supplemental Tables S2 and S3). In addition, all viruses characterized here encoded M1-30D and -215A, which increase H5N1 pathogenicity in mice relative to control viruses [63]. The M viral RNA segment also encodes the ion channel M2 protein, the target of the ion channel inhibitors amantadine and rimantadine. Several amino acid changes in the ion channel confer resistance to ion channel inhibitors [64,65,66,67,68], but the viruses characterized here did not encode any of these amino acids, suggesting that they are sensitive to these drugs. 

NS1 is a multifunctional viral protein that counteracts the innate immune response of the host, enabling the virus to replicate effectively during infection [69]. All viruses analyzed here lacked NS1 amino acids 80–84 (NS1Δ80-84) (Supplemental Tables S2 and S3), a deletion that increases the pathogenicity of H5N1 viruses in mice [70]. The amino acids at positions 103 and 106 (equivalent to positions 98 and 101 in NS1Δ80-84) are important for binding to CPSF30 [71,72], which affects the nuclear export of poly(A)-containing mRNA and thus influenza virus-mediated shutdown of host cell protein synthesis. The H5N1 and H5N6 viruses analyzed in our study encoded NS1-103(98)L/106(101)L (numbers in parentheses refer to the amino acid positions in NS1Δ80-84), resulting in efficient binding to CPSF30. At position 205(200), the viruses of clades 2.3.4.4f and 2.3.4.4g possessed a serine residue, which enhances type I IFN antagonism and pathogenicity in ferrets relative to NS1-205(200)N [73], the residue encoded by clade 2.3.2.1c viruses (Supplemental Tables S2 and S3). Except for individual viruses in clades 2.3.2.1c and 2.3.4.4g, the viruses analyzed in our study possessed the so-called PDZ domain-binding motif “ESEV” at positions 227–230 (222–225), which confers high pathogenicity to influenza virus in mice [74] (Supplemental Tables S2 and S3). Moreover, all viruses characterized here encoded NS1-42S, which confers increased pathogenicity in mice compared with viruses encoding proline at this position [75].

### 3.4. Analysis of Viral Subpopulations

Our analysis of viral subpopulations focused on sequence variants detected with a minimum frequency of 1% at the respective position (Supplemental Table S4). We focused on non-synonymous polymorphisms, that is, sequence variants that resulted in amino acid changes. Samples with ‘mixed’ HA and/or NA genes (indicative of infection with two different viruses) were eliminated from the analysis. With these parameters, no non-synonymous polymorphisms were detected for PB1-F2 and NS2. Polymorphisms in PA, PA-X, and NP were mainly from A/duck/Vietnam/HN4042/2017 (with a frequency of 1–3%) (Supplemental Table S4).

Several non-synonymous polymorphisms were detected in the HA genes (Supplemental Table S4), some of which may affect the biological properties of the viruses. These include sites that form the rim of the receptor-binding domain (i.e., an HA-A125Y polymorphism detected for A/Muscovy duck/Vietnam/HN3790/2017), the receptor-binding pocket (i.e., an HA-R223H polymorphism detected for A/duck/Vietnam/HN3668/2017), the HA cleavage site (i.e., an HA-R327K polymorphisms detected for A/duck/Vietnam/HN4231/2017), or the HA transmembrane domain (i.e., an HA-A523T polymorphism detected for A/duck/Vietnam/HN4231/2017 and A/duck/Vietnam/HN4240/2017). Two viruses (A/Muscovy duck/Vietnam/QN3253/2016 and A/Muscovy duck/Vietnam/QN3262/2016) encoded HA-A134S polymorphisms in 33.6% and 8.4% of sequence reads, respectively. This position is located in the 130-loop, and the A-to-T substitution has been shown to increase binding to α2,6-linked sialic acids [76]. 

Several non-synonymous polymorphisms were detected in the polymerase and NP genes and could affect viral replication. At amino acid position PB2-178, A/Muscovy duck/Vietnam/HN3656/2017 encoded threonine in 78.8% of sequence reads and alanine in 21.2% of sequence reads (Supplemental Table S4); interestingly, we previously found that amino acid changes at this position can affect virus replication (unpublished). A valine-to-isoleucine substitution at position 591 of PB1 contributes to the temperature-sensitive phenotype of the live attenuated influenza A/Leningrad/134/17/57 strain [77]. Here, we detected a PB1-V591D subpopulation in 4.1% of sequence reads of A/duck/Vietnam/HN3680/2017 (Supplemental Table S4). PB1-A661T also contributes to the temperature-sensitive phenotype of the live attenuated influenza A/Leningrad/134/17/57 strain [78]; this amino acid polymorphism was detected in 2.3% of A/duck/Vietnam/HN4231/2017 sequence read (Supplemental Table S4). A/Muscovy duck/Vietnam/HN3801/2017 possessed a PB1-A652V subpopulation (Supplemental Table S4); this amino acid change has been detected in a mouse-adapted human pandemic H1N1 virus [79]. Methionine at PA-336, which confers the high replicative ability to pandemic H1N1 viruses [54], was detected in our study in a small subpopulation of A/Muscovy duck/Vietnam/HN3810/2017 sequence reads (Supplemental Table S4). NP-I365V confers decreased polymerase activity to the H7N9 virus [80] and was detected in 1.6% of sequence reads of A/duck/Vietnam/HK4042/2017 (Supplemental Table S4).

In addition to the polymerase and NP genes, an M1-A37T polymorphism was detected for 1.6% of A/duck/Vietnam/HN3668/2017 sequence reads (Supplemental Table S4); interestingly, the M1-T37A mutation has been reported to increase M1 stability [81] and the infectivity of an H9N2 virus [62]. A/duck/Vietnam/HN3668/2017 also encoded an M2-V27I subpopulation (Supplemental Table S4); this amino acid substitution confers resistance to adamantine [82].

### 3.5. Pathogenicity of H5N1 and H5N6 Viruses in BALB/c Mice

**Figure 3:**
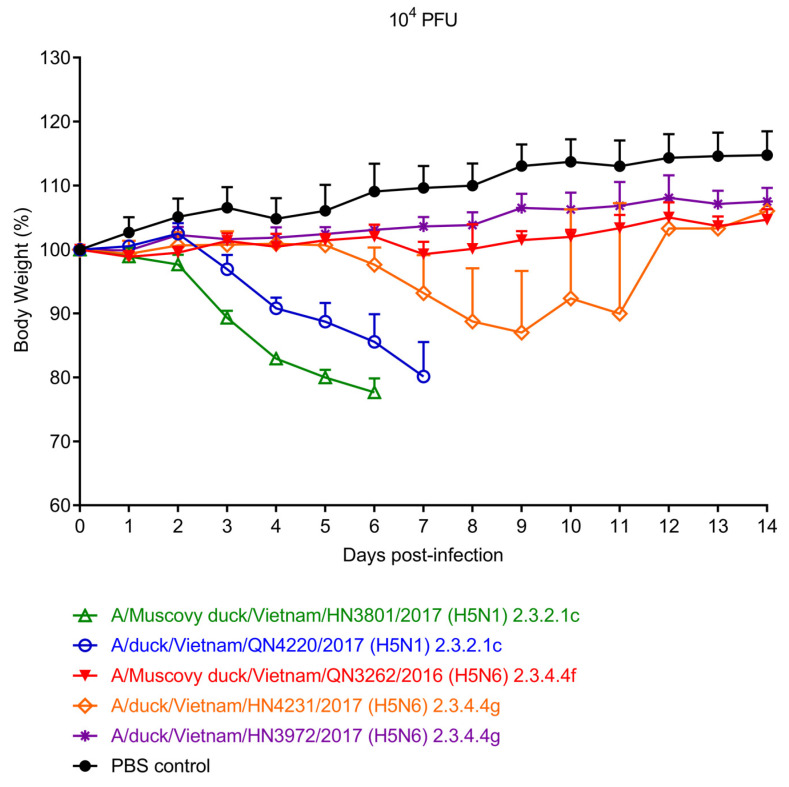
Pathogenicity of representative clade 2.3.2.1c, 2.3.4.4f, and 2.3.4.4g viruses in BALB/c mice. Four mice per group were inoculated intranasally with 10^4^ PFU of the indicated virus and monitored daily for weight loss and mortality. The results shown are mean values ± standard deviations (error bars) from four individual mice.

Five viruses (two from clades 2.3.2.1c and 2.3.4.4g and one from clade 2.3.4.4f) were tested for their pathogenicity in mice by intranasally infecting groups of four 6-week-old BALB/c mice with 10^4^ PFU/50 μL of virus. Mortality and body weight were monitored daily for 14 days. Mice infected with viruses of clade 2.3.2.1c lost body weight rapidly and died or had to be euthanized due to body weight loss of more than 25% of the initial weight within 5 to 7 days of infection (Figure 3). In contrast, the clade 2.3.4.4f and 2.3.4.4g viruses caused no or moderate weight loss and all animals recovered from the virus infection (Figure 3).

## 4. Discussion

Vietnam is one of the countries most affected by outbreaks of highly pathogenic H5 influenza viruses in poultry; in fact, these viruses are now enzootic in poultry populations throughout the country. Live bird markets, as one of the main places for poultry trading, are often the focus of active avian influenza virus surveillance activities. In this study, 27 highly pathogenic H5 influenza viruses were isolated from Vietnamese live bird markets from 2016 to 2017. The isolated viruses belonged to clades 2.3.2.1c, 2.3.4.4f, and 2.3.4.4g, consistent with previous reports on the distribution of clades 2.3.2.1c and 2.3.4.4 viruses mainly in northern Vietnam [83] where our surveillance was conducted. 

Both clade 2.3.2.1c viruses tested in our study were lethal for mice. In contrast, mice survived infection with the clade 2.3.4.4f and 2.3.4.4g viruses tested. Previously, we compared several clades 2.3.2.1a, 2.3.2.1c, 2.3.4.1, and 1.1.2 viruses isolated in Vietnam from 2010 to 2013 and also found that the clade 2.3.2.1 viruses were more pathogenic in mice than clade 2.3.4.1 and clade 1.1.2 viruses [57]. Collectively, these data may indicate that clade 2.3.2.1 viruses are more pathogenic in mice than viruses from other clades. The clade 2.3.2.1c viruses tested here differ from the clade 2.3.4.4f and clades 2.3.4.4g viruses in several genetic features including an N1 NA (all clade 2.3.4.4f and clade 2.3.4.4g viruses possessed N6 NA), deletion of amino acids 49–68 in N1, which is known to increase pathogenicity (reviewed in [28]), and a truncated PB1-F2 of 57 amino acids (with all clade 2.3.4.4f and clade 2.3.4.4g viruses encoding full-length PB1-F2 proteins). We currently do not know which of these differences (or combinations thereof) may confer higher pathogenicity to clade 2.3.2.1c viruses compared with clade 2.3.4.4f and clade 2.3.4.4g viruses. 

HPAI H5 viruses have evolved rapidly through reassortment and mutations [16,84]. Here, we also found that some of the viruses characterized possessed genes that are most closely related to low pathogenic avian influenza viruses. For example, some of the PB2 genes were most closely related to low pathogenic avian H9N2 viruses, which are known to share gene segments encoding internal proteins with H5, H7N9, H10N8, and H3N8 viruses. Thus, despite attempts to control HPAI H5 viruses, they continue to reassort with low pathogenic avian influenza viruses, which may alter their phenotypes. In summary, our avian influenza virus surveillance in Vietnam resulted in the isolation of several HPAI viruses, some of which are lethal in mice and possess mutations that may affect their pathogenicity.

## Figures and Tables

**Table 1:** H5 avian influenza viruses isolated in Vietnamese live bird markets in 2016–2017.

Virus	Subtype(s) *	Subclade	Location	Province	Region of Vietnam	Date Collected
A/duck/Vietnam/HN3443/2016	H5/H6, N1/N6	2.3.2.1c	Thanh Tri	Ha Noi	North	5 December 2016
A/duck/Vietnam/HN3448/2016	H5/H6, N1/N6	2.3.2.1c	Thanh Tri	Ha Noi	North	5 December 2016
A/Muscovy duck/Vietnam/HN3506/2017	H5, N1/N6	2.3.2.1c	Thanh Tri	Ha Noi	North	6 January 2017
A/Muscovy duck/Vietnam/HN3590/2017	H5N1	2.3.2.1c	Thanh Tri	Ha Noi	North	15 February 2017
A/Muscovy duck/Vietnam/HN3656/2017	H5N1	2.3.2.1c	Thuong Tin	Ha Noi	North	10 March 2017
A/Muscovy duck/Vietnam/HN3790/2017	H5N1	2.3.2.1c	Gia Lam	Ha Noi	North	24 April 2017
A/Muscovy duck/Vietnam/HN3801/2017	H5N1	2.3.2.1c	Thuong Tin	Ha Noi	North	10 May 2017
A/Muscovy duck/Vietnam/HN3810/2017	H5N1	2.3.2.1c	Thuong Tin	Ha Noi	North	10 May 2017
A/duck/Vietnam/QN4220/2017	H5N1	2.3.2.1c	Ha Long	Quang Ninh	North	26 October 2017
A/Muscovy duck/Vietnam/QN3253/2016	H5N6	2.3.4.4f	Cao Xanh	Quang Ninh	North	13 September 2016
A/Muscovy duck/Vietnam/QN3262/2016	H5N6	2.3.4.4f	Ha Long	Quang Ninh	North	13 September 2016
A/Muscovy duck/Vietnam/QN3318/2016	H3/H5, N2/N6	2.3.4.4f	Ha Long	Quang Ninh	North	11 October 2016
A/duck/Vietnam/QN3328/2016	H5/H6, N6/N8	2.3.4.4f	Ha Long	Quang Ninh	North	11 October 2016
A/duck/Vietnam/QN3335/2016	H3/H5, N6	2.3.4.4f	Cao Xanh	Quang Ninh	North	11 October 2016
A/Muscovy duck/Vietnam/HN3515/2017	H5/H6, N6	2.3.4.4f	Thanh Tri	Ha Noi	North	6 January 2017
A/duck/Vietnam/QN3556/2017	H5, N1/N6	2.3.4.4f	Dong Trieu	Quang Ninh	North	21 January 2017
A/Muscovy duck/Vietnam/HN3667/2017	H5N6	2.3.4.4f	Thuong Tin	Ha Noi	North	10 March 2017
A/duck/Vietnam/HN3668/2017	H5N6	2.3.4.4f	Thuong Tin	Ha Noi	North	10 March 2017
A/duck/Vietnam/HN3680/2017	H5N6	2.3.4.4f	Thuong Tin	Ha Noi	North	10 March 2017
A/Muscovy duck/Vietnam/HN3943/2017	H5/H6, N6	2.3.4.4g	Thuong Tin	Ha Noi	North	10 July 2017
A/Muscovy duck/Vietnam/HN3948/2017	H5/H6, N6	2.3.4.4g	Thuong Tin	Ha Noi	North	10 July 2017
A/duck/Vietnam/HN3972/2017	H5N6	2.3.4.4g	Gia Lam	Ha Noi	North	12 July 2107
A/duck/Vietnam/HN3977/2017	H5N6	2.3.4.4g	Gia Lam	Ha Noi	North	12 July 2107
A/duck/Vietnam/HN4042/2017	H5N6	2.3.4.4g	Gia Lam	Ha Noi	North	10 August 2017
A/duck/Vietnam/HN4043/2017	H5N6	2.3.4.4g	Gia Lam	Ha Noi	North	10 August 2017
A/duck/Vietnam/HN4231/2017	H5N6	2.3.4.4g	Thuong Tin	Ha Noi	North	12 November 2017
A/duck/Vietnam/HN4240/2017	H5N6	2.3.4.4g	Thuong Tin	Ha Noi	North	12 November 2017

* For ‘mixed’ samples, both HA and/or NA subtypes are listed.

## Data Availability

All sequences were deposited to GenBank with the following accession numbers: OQ546728 to OQ546795; OQ546869 to OQ546921; OQ547053 to OQ547096; OQ596733 to OQ596736; OQ596768 to OQ596783; OQ596785 to OQ596800; OQ596802 to OQ596819; and OQ600194; OQ603031 to OQ603034. Data output from the NGS sequence analysis pipeline are available in Supplemental Tables S2 and S3.

## Supplementary Materials

The following supporting information can be downloaded at: https://www.mdpi.com/article/10.3390/v15051093/s1, Figure S1: Maximum-likelihood (ML) phylogeny of N1 NA genes; Figure S2: Maximum-likelihood (ML) phylogeny of N6 NA genes; Figure S3: Maximum-likelihood (ML) phylogeny of PB2 genes; Figure S4 Maximum-likelihood (ML) phylogeny of NS1 genes; Table S1: GISAID acknowledgements for H5 HA sequences; Table S2: Consensus amino acid sequences of isolated H5 viruses; Table S3: Summary of identified SNPs.
