# Supplementary material for: Highly Pathogenic H5 Influenza Viruses Isolated between 2016 and 2017 in Vietnamese Live Bird Markets
Source: Viruses. 2023 Apr 29;15(5):1093. doi: 10.3390/v15051093 (PMC10223276; doi:10.3390/v15051093)
Supplement: Supplementary file 1 [file viruses-15-01093-s001.zip › Supplemental figure legends.pdf]

Figure S1. Maximum-likelihood (ML) phylogeny of N1 NA genes. Viruses isolated in this study are shown in purple (clade 2.3.2.1c) and red (clade 2.3.4.4f). Bootstrap values greater than 80% are indicated at the nodes. The scale bar represents the nucleotide substitution rate at each site.

Figure S2. Maximum-likelihood (ML) phylogeny of N6 NA genes. Viruses isolated in this study are shown in purple (clade 2.3.2.1c), red (clade 2.3.4.4f), and blue (clade 2.3.4.4g). Bootstrap values greater than 80% are indicated at the nodes. The scale bar represents the nucleotide substitution rate at each site.

Figure S3. Maximum-likelihood (ML) phylogeny of PB2 genes. Viruses isolated in this study are shown in purple (clade 2.3.2.1c), red (clade 2.3.4.4f), and blue (clade 2.3.4.4g). Bootstrap values greater than 80% are indicated at the nodes. The scale bar represents the nucleotide substitution rate at each site.

Figure S4. Maximum-likelihood (ML) phylogeny of NS1 genes. Viruses isolated in this study are shown in purple (clade 2.3.2.1c), red (clade 2.3.4.4f), and blue (clade 2.3.4.4g). Bootstrap values greater than 80% are indicated at the nodes. The scale bar represents the nucleotide substitution rate at each site.
