# Supplementary figures and images for: Highly Pathogenic H5 Influenza Viruses Isolated between 2016 and 2017 in Vietnamese Live Bird Markets
Source: Viruses. 2023 Apr 29;15(5):1093. doi: 10.3390/v15051093 (PMC10223276; doi:10.3390/v15051093)

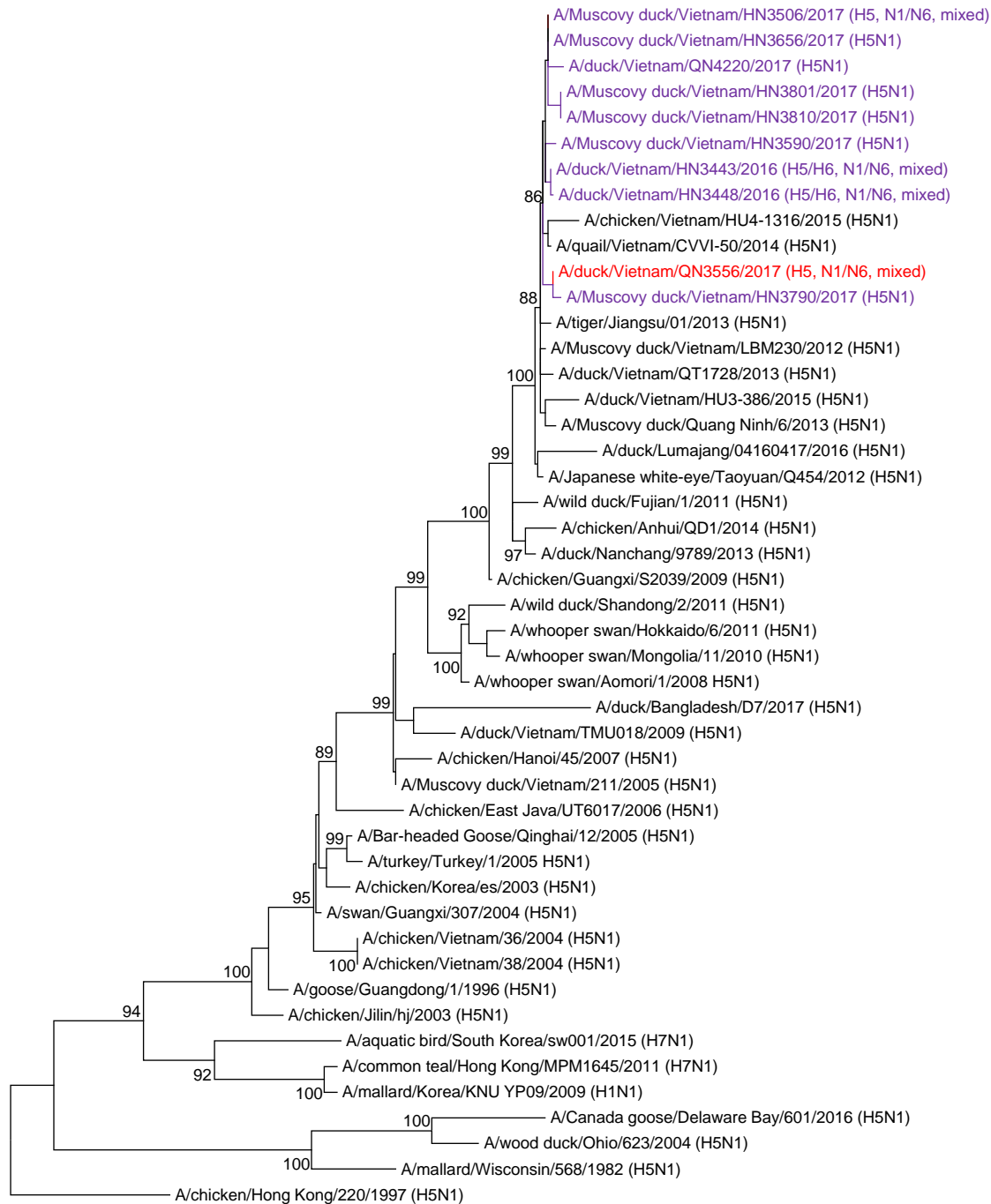

Figure S1. N1 NA gene

Supplement: Supplementary file 1 [file viruses-15-01093-s001.zip › Guan - VN Surveillance - Figure S1.pdf]

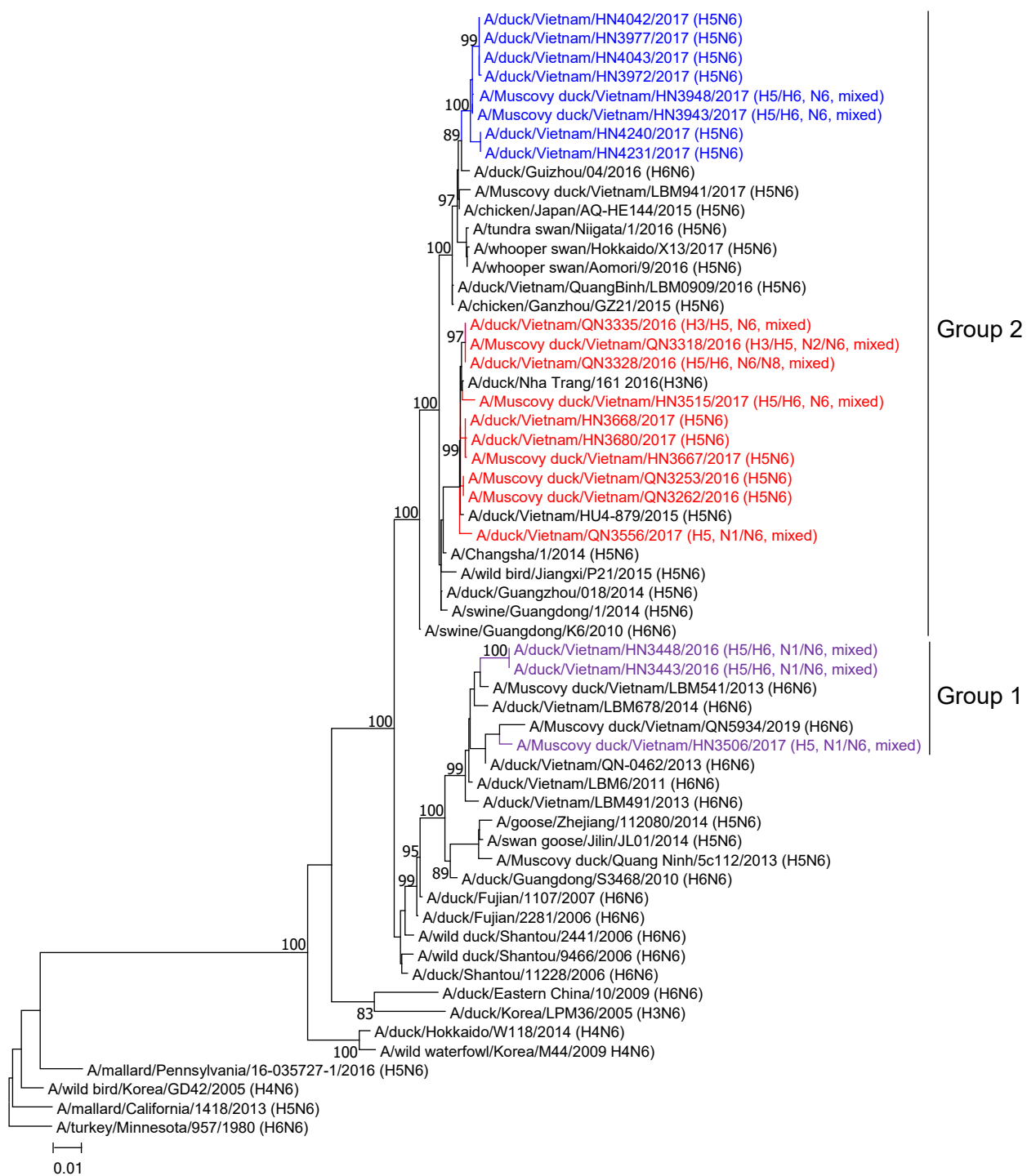

Figure S2. N6 NA gene

Supplement: Supplementary file 1 [file viruses-15-01093-s001.zip › Guan - VN Surveillance - Figure S2.pdf]

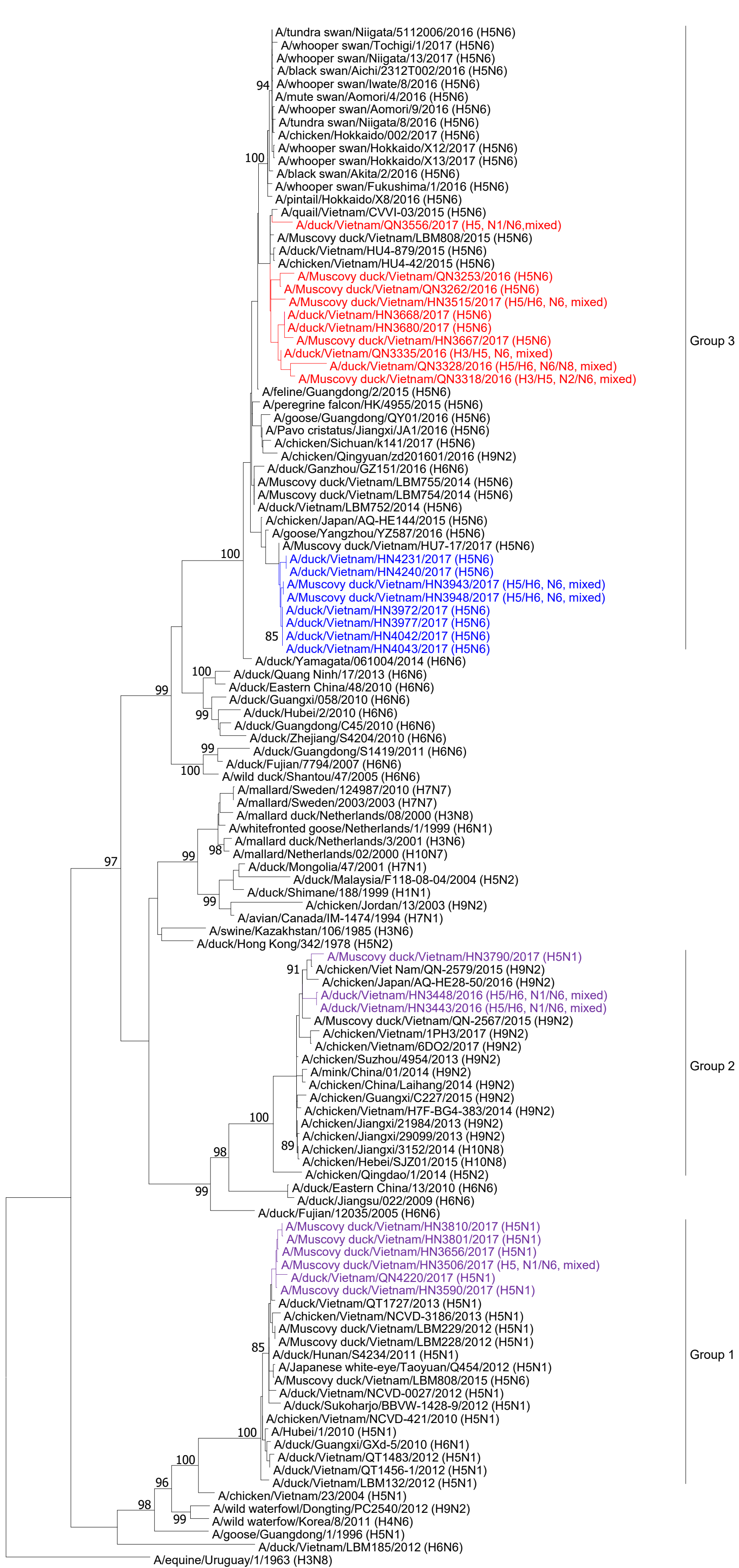

0.01

Supplement: Supplementary file 1 [file viruses-15-01093-s001.zip › Guan - VN Surveillance - Figure S3.pdf]

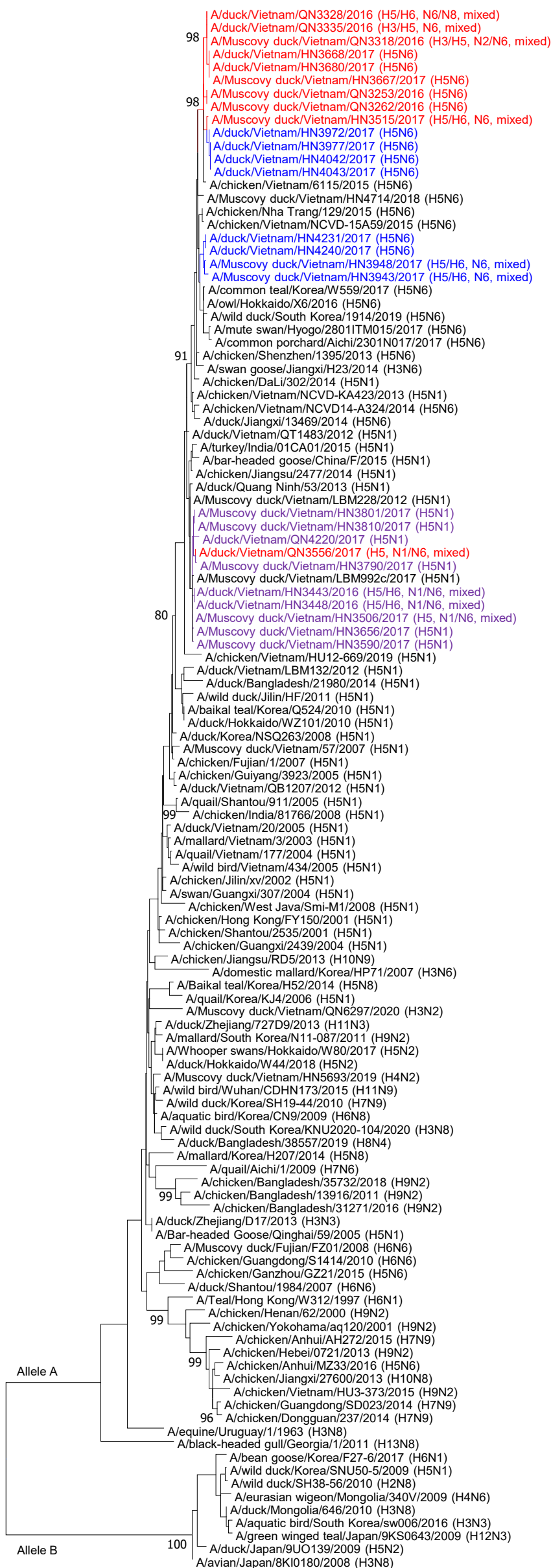

Supplement: Supplementary file 1 [file viruses-15-01093-s001.zip › Guan - VN Surveillance - Figure S4.pdf]
